# All together to Fight COVID-19

**DOI:** 10.4269/ajtmh.20-0281

**Published:** 2020-04-22

**Authors:** Sara Momtazmanesh, Hans D. Ochs, Lucina Q. Uddin, Matjaz Perc, John M. Routes, Duarte Nuno Vieira, Waleed Al-Herz, Safa Baris, Carolina Prando, Laszlo Rosivall, Amir Hamzah Abdul Latiff, Timo Ulrichs, Vasili Roudenok, Juan Carlos Aldave Becerra, Deepak B. Salunke, Ekaterini Goudouris, Antonio Condino-Neto, Anzhela Stashchak, Oleksandr Kryvenko, Mykola Stashchak, Anastasia Bondarenko, Nima Rezaei

**Affiliations:** 1 School of Medicine, Tehran University of Medical Sciences, Tehran, Iran;; 2 Universal Scientific Education and Research Network (USERN), The World;; 3 Department of Pediatrics, University of Washington, Seattle, Washington;; 4 Seattle Children’s Research Institute, Seattle, Washington;; 5 Department of Psychology, University of Miami, Coral Gables, Florida;; 6 Faculty of Natural Sciences and Mathematics, University of Maribor, Maribor, Slovenia;; 7 Department of Medical Research, China Medical University Hospital, China Medical University, Taichung, Taiwan;; 8 Department of Pediatrics, Medical College of Wisconsin, Milwaukee, Wisconsin;; 9 University of Coimbra, Faculty of Medicine, Coimbra, Portugal;; 10 Institute of Legal Medicine and Institute of Bioethics, Coimbra, Portugal;; 11 Department of Pediatrics, Faculty of Medicine, Kuwait University, Kuwait City, Kuwait;; 12 Division of Pediatric Allergy/Immunology, Marmara University Hospital, Marmara University, Istanbul, Turkey;; 13 Instituto de Pesquisa Pelé Pequeno Príncipe, Curitiba, Brazil;; 14 Faculdades Pequeno Príncipe, Curitiba, Brazil;; 15 Institute of Translational Medicine, International Nephrology Research and Training Center, Faculty of Medicine, Semmelweis University, Budapest, Hungary;; 16 Allergy and Immunology Centre, Pantai Hospital Kuala Lumpur, Kuala Lumpur, Malaysia;; 17 Institute for Research in International Assistance, Akkon University for Human Sciences, Berlin, Germany;; 18 Belarusian State Medical University, Minsk, Belarus;; 19 Hospital Nacional Edgardo Rebagliati Martins, Lima, Peru;; 20 Panjab University (PU), Chandigarh, India;; 21 Pediatrics Department, Medical School, Federal University of Rio de Janeiro, Rio de Janeiro, Brazil;; 22 Department of Immunology, Institute of Biomedical Sciences, University of São Paulo, São Paulo, Brazil;; 23 Kharkiv National Medical University, Kharkiv, Ukraine;; 24 Pediatric Infectious Disease and Pediatric Immunology Department, Shupyk National Medical Academy for Postgraduate Education, Kiev, Ukraine

## Abstract

Novel coronavirus disease (COVID-19), named a pandemic by the WHO, is the current global health crisis. National and international collaboration are indispensable for combating COVID-19 and other similar potential outbreaks. International efforts to tackle this complex problem have led to remarkable scientific advances. Yet, as a global society, we can and must take additional measures to fight this pandemic. Undoubtedly, our approach toward COVID-19 was not perfect, and testing has not been deployed fast enough to arrest the epidemic early on. It is critical that we revise our approaches to be more prepared for pandemics as a united body by promoting global cooperation and commitment.

In December 2019, novel coronavirus disease (COVID-19) emerged in Wuhan, China, attracting the notice of regional authorities and rapidly drawing global attention. According to the WHO, in less than 4 months, COVID-19 spread through almost all countries and regions. The COVID-19 pandemic is wreaking havoc on the world economy, in addition to creating the current global health crisis.^1^

In recent decades, we have witnessed several other epidemic outbreaks, which have highlighted the importance of strengthening our ability to anticipate epidemics and pandemics in addition to taking appropriate and timely global measures. The most prominent recent outbreaks included severe acute respiratory syndrome in 2003, H1N1 influenza in 2009, Middle East respiratory syndrome in 2012, and Ebola in West Africa in 2014–2016.^2^ Every one of these experiences taught us salutary lessons for managing similar situations in the future and reminded us that we remain inadequately prepared for a pandemic.^3^ One of these invaluable lessons was that international collaboration is an indispensable part of the preparation for such outbreaks. We have made significant progress in establishing global guidelines to prevent epidemics and pandemics. The establishment of several organizations supported by the WHO to coordinate international measures for pandemic threats is an example of these endeavors.^4^ The “Coalition for Epidemic Preparedness Innovations” and “Research Collaboration for Infectious Disease Preparedness” are examples of such organizations that are currently actively looking for potential therapies and vaccines for COVID-19.

Our rapidly advancing knowledge of COVID-19, which was gained only through extensive worldwide scientific collaboration, is a showcase of the international efforts to tackle this problem. One of the initial effective actions was the rapid sharing of the genomic sequence of COVID-19, which enabled numerous researchers around the world to study this virus simultaneously.^5^ In addition, rapid publishing of data relevant to COVID-19 set the stage for researchers to work on this still-evolving challenge worldwide. As of mid-April 2020, nearly 4,000 articles addressing COVID-19 were registered in PubMed, sharing clinical and epidemiological features of the disease, results of in vitro and in vivo investigations aimed at finding effective treatments and vaccines, and other advances. An important factor promoting advances on COVID-19 was the provision of open access to reports on this topic by many publishers. A remarkable collaborative activity was the launch of the Solidarity trial, which aims to find an effective treatment for COVID-19 and is currently being conducted in at least 10 countries, by the WHO.^6^

In addition to scientific accomplishments, national and international cooperation have helped tremendously in curbing the spread of the virus. On a national level, many countries are educating the public with reliable and accurate data and instructions, provided by both political and scientific authorities.^7^ Compliance of citizens with policies such as social distancing, sanitation, and isolation is playing a major role in controlling this pandemic. Understanding the enormous impact of these policies and practices on individual and public health is key to maintaining the public’s compliance. On a global scale, since the beginning of the pandemic, the WHO shared COVID-19 testing kits and personal protective equipment (PPE) with more than 120 countries.^8^ It also imposed interim guidance for testing and diagnosis,^9^ unifying testing methods around the world to facilitate detection of infected individuals.

There are still many additional collaborative measures we can take as a united body in combating COVID-19, particularly in the scientific field. First, global collaboration can facilitate multisite randomized clinical trials to explore effective diagnostics, drugs, and potential vaccines, bringing together industry, academia, and public health entities to expedite rapidly bringing products to the public. Second, both on the national and the international level, the continuous exchange of knowledge and experience among healthcare providers who are on the front line in the fight against COVID-19 will be extremely constructive. Third, considering the role of the host immune system in fighting COVID-19^10^ and reports of some families with severe forms of COVID-19,^11^ collaborative genetic studies to identify genetic defects associated with poor outcomes will help to characterize disease pathogenesis. Fourth, studies to determine the psychological and neurological impact of the virus on those who have been infected will be helpful. Importantly, scientific advances need to be shared rapidly, as was the case with the determination of the viral sequence early in 2020. When we identify an improved clinical algorithm or find an effective diagnostic treatment or vaccine, all countries, either developed or developing, should have prompt and adequate access.

It is important to learn from our experience with COVID-19, including flaws in our approach, to try to organize policies to avoid such errors in the future when we face another potential pandemic. With the quick spread of COVID-19 across the globe, we have to admit that our measures were not sufficient, and also not deployed fast enough, to arrest the epidemic early on. Before the occurrence of COVID-19, several reports described our lack of preparedness for such crises. The Global Preparedness Monitoring Board (GPMB) is “an independent monitoring and accountability body to ensure preparedness for global health crises co-convened by the WHO and the World Bank.”^12^ In its annual report on global preparedness for health emergencies, the GPMB recently stated that the world has not done enough to prepare against emerging pandemics. They recommended several solutions, including full compliance with the International Health Regulations (IHR), a legal commitment of countries to prevent the spread of disease^13^; support for the poorest countries to prepare against these global threats; and anticipation of potential pandemics before they pose a severe risk.^2^

Here, we make other suggestions to allow us to be more prepared for such potential disasters as a global society, with a focus on learning from our mistakes during the COVID-19 pandemic. In the initial stages, when the extent to which the virus was contagious and dangerous was not clear, the outbreak was underestimated by the global society. As a result, international travel, which was the main cause of international spread of the virus, was not adequately restricted. There was a substantial delay in calling COVID-19 a public health emergency of international concern (PHEIC), and finally a pandemic. When we are dealing with such a contagious respiratory microorganism, even before it spreads to many countries, we need to know that it is a potential pandemic and to deploy the same guidelines we apply during a pandemic, the most important one of which is isolation. Detecting microorganisms which can potentially create pandemics based on their epidemiological features before they infect many countries and promptly reducing international travel will help tremendously in slowing down the spread of viral outbreaks.

In the next stage, after the announcement of COVID-19 as a PHEIC, many countries did not follow international regulations and guidelines, particularly the IHR. This was in part due to the fact that these organizations do not have adequate executive power to enforce public health guidance. Establishment of the so-called World Organization for Pandemic Anticipation and Prevention, jointly by the United Nations, as the executive body, and the WHO, as the expert organization, can limit the recurrence of similar crises through timely recognition and intervention by all member countries.

When almost all countries faced the challenge of combating COVID-19 and experienced its detrimental repercussions, we faced a global lack of testing equipment, PPE, intensive care unit beds, and other resources. All of these limitations accelerated the spread of the virus, the most deleterious one of which was limited reliable testing kits. As we saw around the world with COVID-19, delays in detecting infected individuals can have dire consequences. Providing affordable, rapid, reliable testing methods to every country should be a high priority in similar situations. The global shortage of PPE also speeded up the spread of the virus, especially to healthcare providers. To avoid recurrence of this tragedy, large quantities of PPE should always be on hand, and once an outbreak with pandemic potential is recognized, there should be rapid scale-up of production.

To conclude, COVID-19 is testing our preparedness for fighting pandemics, a key element of which is national and international cooperation. We have made considerable progress in this fight. However, we can learn from our mistakes during our recent experience with COVID-19. The more we delay the implementation of appropriate actions, the more extraordinary measures need to be used, and the more human suffering increases. Infectious disease outbreaks must be announced immediately, and policies for tackling them should be made as quickly as possible. It is critical that we revise our approaches to become more prepared for pandemics by promoting global cooperation and commitment.
